# A novel bilayered expanded polytetrafluoroethylene glaucoma implant creates a permeable thin capsule independent of aqueous humor exposure

**DOI:** 10.1002/btm2.10179

**Published:** 2020-08-22

**Authors:** Amanda Kiely Bicket, Julia Szeto, Peter Roeber, Jeff Towler, Mitch Troutman, E. Randy Craven, Anup Khatana, Ike Ahmed, Harry Quigley, Pradeep Ramulu, Ian F. Pitha

**Affiliations:** ^1^ Wilmer Eye Institute, Johns Hopkins University School of Medicine Baltimore Maryland USA; ^2^ W.L. Gore & Associates Newark Delaware USA; ^3^ Cincinnati Eye Institute Cincinnati Ohio USA; ^4^ Department of Ophthalmology and Vision Sciences University of Toronto Toronto Canada

**Keywords:** bleb, capsule, expanded polytetrafluoroethylene (ePTFE), fibrosis, glaucoma, glaucoma drainage implant (GDI), intraocular pressure (IOP)

## Abstract

The purpose of these studies was to evaluate clinical, functional, and histopathological features of glaucoma drainage implants (GDIs) fabricated from novel, custom‐tailored expanded polytetrafluoroethylene (ePTFE). Implants of matching footprints were fabricated from silicone (Control) and novel, bilayered ePTFE. ePTFE implants included: (a) one that inflated with aqueous humor (AH) (High), (b) one that inflated with a lower profile (Low), (c) an uninflated implant not connected to the anterior chamber (Flat), and (d) one filled with material that did not allow AH flow (Filled). All implants were placed in adult New Zealand White rabbits and followed over 1–3 months with clinical exams and intraocular pressure. The permeability of tissue capsules surrounding GDIs was assessed using constant‐flow perfusion with fluoresceinated saline at physiologic flow rates. After sacrifice, quantitative histopathological measures of capsule thickness were compared among devices, along with qualitative assessment of cellular infiltration and inflammation. Capsular thickness was significantly reduced in blebs over ePTFE (61.4 ± 53 μm) versus silicone implants (193.6 ± 53 μm, *p* = .0086). AH exposure did not significantly alter capsular thickness, as there was no significant difference between High and Filled (50.9 ± 29, *p* = .34) implants. Capsules around ePTFE implants demonstrated permeability with steady‐state pressure: flow relationships at physiologic flow rates and rapid pressure decay with flow cessation, while pressure in control blebs increased even at low flow rates and showed little decay. Perfused fluorescein dye appeared beyond the plate border only in ePTFE implants. ePTFE implants are associated with thinner, more permeable capsules compared to silicone implants simulating presently used devices.

## 1 INTRODUCTION

There has been renewed interest in improving the success and complication rate of incisional glaucoma surgery. Commonly used approaches create an outlet for aqueous humor (AH) release from the anterior chamber to the subconjunctival space through transscleral techniques (trabeculectomy, deep sclerectomy) or by inserting an implant (glaucoma drainage implant [GDI], XEN Gel Stent [Allergan], PreserFlo Microshunt [Santen]) that facilitates AH release to a subconjunctival tissue pocket (a bleb). Failure rates due to inadequately controlled intraocular pressure (IOP) or need for reoperation have been reported as high as 46.9% over 5 years for trabeculectomy,^1^ and the equivalent rates for Ahmed and Baerveldt GDIs are 32 and 18%, respectively.^2^ Complications of incisional surgeries include excessive IOP reduction (hypotony), diplopia, bleb leaks, device extrusion, and infection.^3^ The 5‐year rate of erosion for GDIs has been reported between 1 and 5%.^3^, ^4^ Risk of wound leak following trabeculectomy is significant as well, reported as 6% over 5 years.^3^

Inadequate IOP reduction following incisional glaucoma surgeries most commonly results from loss of bleb capsule permeability to AH flow (also referred to as capsular porosity or hydraulic conductivity) due to excessive fibrosis.^5^, ^6^ Bleb capsule thickness and permeability are potentially affected by several factors, including individual patient healing rate, the actions of cytokines and anti‐proliferative factors present in AH, and characteristics of the implant itself.^7^, ^8^, ^9^, ^10^, ^11^, ^12^, ^13^ Wilcox et al. showed that larger GDI plate radius led to thicker capsules.^14^, ^15^ They hypothesized that the greater height of a larger bleb increases capsule tension as modeled by Laplace's law, which leads to collagen deposition and capsular thickening.^14^ The influences of bleb geometry and AH exposure were explored further by Coote and coworkers, who showed that outflow resistance around GDIs implanted in rabbit eyes increased with exposure to either AH^16^ or balanced salt solution, suggesting that capsule tension alone, and not AH cytokines, is sufficient to reduce permeability.^17^

Currently, glaucoma surgeons utilize a variety of techniques to prevent excessive fibrosis. The primary technique used in conjunction with trabeculectomy and XEN Gel Stent placement is antifibrotic application at the time of surgery.^18^, ^19^ Mitomycin C application has been associated with improved clinical outcomes, but comes with an increased risk of postoperative infection and hypotony.^20^, ^21^ The benefits of antifibrotic use with GDI placement have not been established.^22^ Surgeons use several ad hoc modifications in order to reduce postoperative fibrosis following GDI placement, such as two‐stage implantation to delay AH flow, or placement of the GDI plate more posteriorly or above the Tenon's capsule.^23^, ^24^ Large‐scale, prospective randomized trials have not been performed to establish the potential benefit of these modifications. A promising and incompletely explored approach to fibrosis control utilizes biomaterials that modulate healing. An ideal biomaterial for use in bleb‐forming glaucoma surgery would (a) incite neither toxicity nor immune response (biocompatibility), (b) permit sufficient healing to minimize risk of exposure and infection, (c) provide optimal permeability of the capsule formed around the device, and (d) abrogate any pro‐inflammatory effects of AH.

Expanded polytetrafluoroethylene (ePTFE) is a highly stable polymer of tetrafluoroethylene that was patented by Gore.^25^ Due to its biocompatibility, biostability, and high compliance, ePTFE incorporates well into many tissues and is approved for use in numerous biomedical implants, including: vascular grafts, bypass grafts, hernia membranes, and sutures. ^26^, ^27^ ePTFE has a porous surface comprised of nodes and fibrils which can structurally permit or prevent cellular integration. We hypothesized that an ePTFE GDI would allow physiologic tissue integration, creating a thinner more permeable capsule while allowing sufficient healing to avoid exposure. We additionally hypothesized that bleb capsule tension would be a dominant factor influencing capsule thickness and permeability. Here, we test these hypotheses by implanting ePTFE GDI devices in rabbits to compare their features to a silicone GDI similar to present devices.

## 2 MATERIALS AND METHODS

### 2.1 Animals

Adult female New Zealand White (NZW) rabbits (3.5–5.0 kg) were used in experimental protocols approved by the Animal Care and Use Review Board of Johns Hopkins University School of Medicine. Rabbits were handled in a manner consistent with the ARVO Statement for the Use of Animals in Ophthalmic and Vision Research, and the Guide for the Care and Use of Laboratory Animals (Institute of Laboratory Animal Research, the Public Health Services Policy on Humane Care and Use of Laboratory Animals).

### 2.2 Implant device materials and construction

Control glaucoma drainage devices were made of 40‐durometer silicone sheeting of 0.25 mm nominal thickness with a smooth finish. A silicone tube with an outer diameter of 0.5 mm and inner diameter of 0.25 mm, chosen to facilitate surgical placement through a 25G needle tract, was attached using a silicone adhesive. Experimental implants devices were fashioned from novel, bilayered ePTFE membranes; two such membranes were continuously bonded to one another around the device outer perimeter to form a central reservoir chamber with a total nominal material thickness of 0.2 mm prior to inflation. ePTFE has a microporous structure comprised of dense “nodes” joined by thin “fibrils”; its pore size refers to the distance between nodes.^28^ A unique aspect of the ePTFE used here was that the membranes were comprised of an exterior “open” layer with a larger pore size into which surrounding cells could integrate, and an inner “tight” layer with pores too small for cellular ingress into the reservoir, but through which AH could pass readily. A silicone tube identical to that used for control devices was sandwiched between the layers and attached in watertight fashion with a silicone adhesive. The preimplantation resistance to flow through experimental devices was negligible at physiologic flow rates. All devices were sterilized by ethylene oxide after manufacturing and packaged in sterilization pouches prior to implantation.

### 2.3 Characterization of implants

**FIGURE 1:**
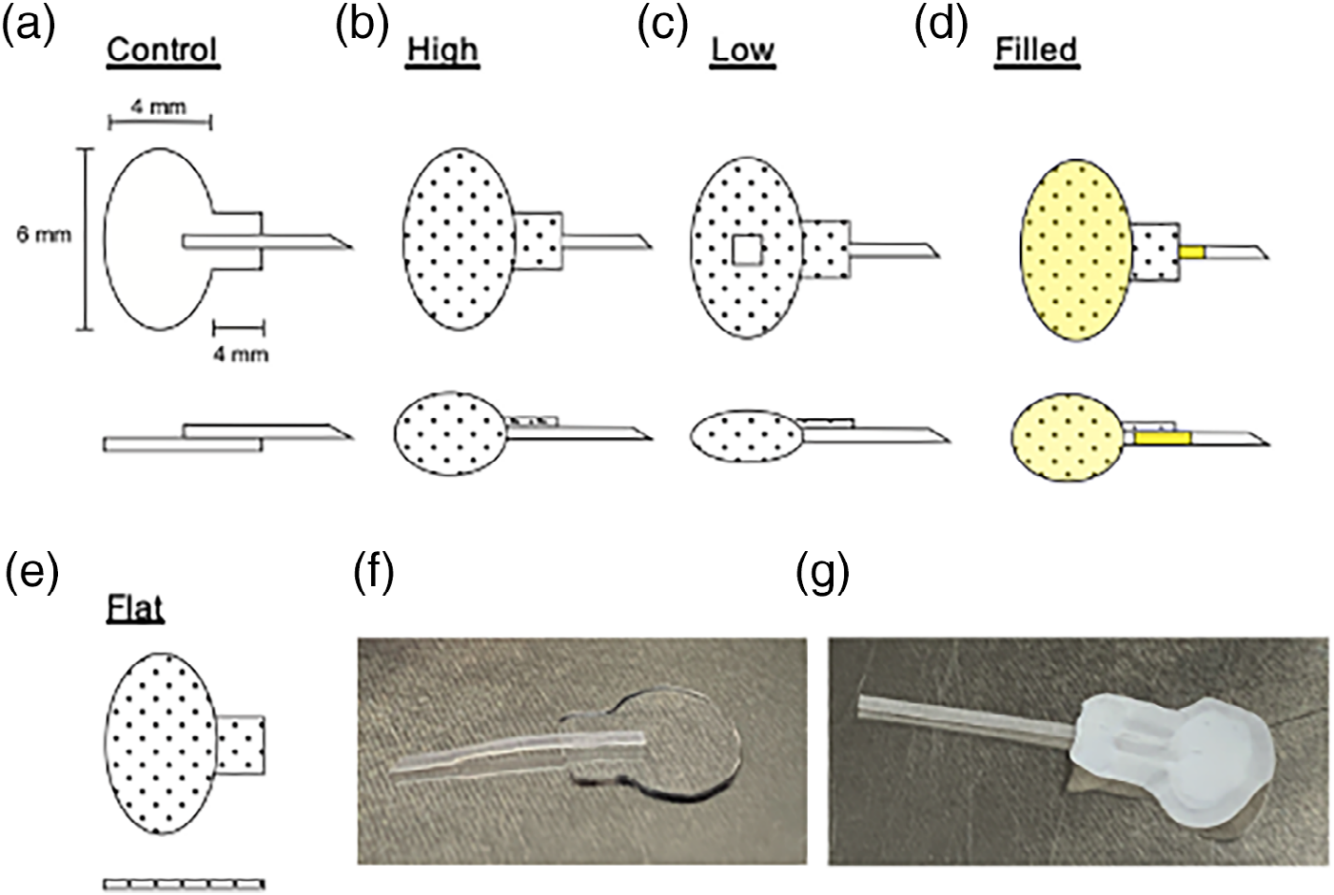
Schematic diagrams and gross images of implants. Footprint (above) and side view (below) of implants (a–e). No fill indicates silicone material, dot fill indicates expanded polytetrafluoroethylene (ePTFE), and yellow fill indicates obstructed tube and material filling *Filled* reservoir. Gross pictures of a control (f) and *High* (g) implant prior to placement in vivo

Five implant designs were evaluated, all with identical two‐dimensional footprints prior to implantation. Implant dimensions were chosen to fit between the extraocular muscles of NZW rabbits and anterior to their posterior orbital venous sinuses. Schematics are shown in Figure 1, and a brief description of experimental protocols, including the number of each implant type placed and duration of follow‐up, is found in Table 1. The five implants were:

1. A Control implant consisting of a flat silicone plate with a silicone tube attached to its outer (conjunctival) side, as in the Baerveldt implant.
2. An ePTFE implant with an inflatable reservoir attached to a silicone tube. In the uninflated state, the footprint of the reservoir was the same as the silicone control; however, AH entry through the tube fully inflated the reservoir (High).
3. An ePTFE implant identical to (2), but with the tube occluded by a silicone plug and the reservoir filled with additional ePTFE material to mimic the shape produced by inflation, without AH exposure (Filled).
4. An ePTFE implant similar to (2), but with an inflatable reservoir whose height was limited by a central rivet (Low).
5. An ePTFE implant with a reservoir identical to (2) but no tube, so that it was neither inflated nor connected to the anterior chamber (Flat). The Flat implant cannot inflate and maintains a flat footprint without AH flow.

**TABLE 1:** Experimental study groups and the number of animals within each

			Number of eyes			
Device name	Description	Aqueous flow	1 month	2 months	3 months	Total
Control	Silicone plate	+	10	8	4	22
High	ePTFE reservoir, unrestricted height	+	9	4	4	17
Low	ePTFE reservoir, height limited by rivet	+	4	0	0	4
Flat	ePTFE reservoir, flat	−	3	0	0	3
Filled	ePTFE reservoir, filled with internal ePTFE	−	4	0	0	4

Abbreviation: ePTFE, expanded polytetrafluoroethylene.

These designs were created in order to test the performance of ePTFE compared to standard silicone plates (control vs. High implants), as well as to isolate the effects of implant height (High vs. Flat and Low) and AH exposure (High and Low vs. Filled) on implant performance and capsule formation.

### 2.4 IOP measurement

IOP was measured with the TonoVet tonometer (TioLat, Inc., Helsinki, Finland) in awake, restrained rabbits, with the magnetic probe moving horizontally, according to the manufacturer's instructions. All measurements were performed by a trained technician, without topical anesthesia. Prior to measurements, each rabbit was restrained and allowed to calm for at least 3 min. Three IOP measurements were taken of each eye; if measurements differed by >2 mmHg, the rabbit was allowed to acclimate for three more minutes before repeat measurements were taken.

### 2.5 Device implantation

All surgeries were performed by two experienced glaucoma surgeons (AB, IP). Rabbits were sedated with intramuscular ketamine (20–45 mg/kg) and xylazine (1–2 mg/kg) (MWI Animal Health, Boise, ID), intubated, and maintained on 0.5–2.3% isoflurane with 1–3 L/min O_2_. Vital parameters including pulse oximetry, heart rate, respiratory rate, end tidal CO_2_, electrocardiogram activity, and body temperature were continuously monitored during the procedure. Topical proparacaine (1%) was placed in the operative eye and the eye and surrounding eyelids were prepped using a sterile betadine solution. A 2 clock‐hour, superotemporal peritomy was made and posterior subconjunctival dissection was used to create a fornix‐based pocket for implant placement. The implant was placed in the pocket resting flat on the scleral surface. A scleral tunnel was made approximately 1.5 mm posterior to the limbus using a 25G needle and the implant's tube inserted into the anterior chamber through the prepared tunnel. AH was noted to fill open ePTFE reservoirs and flow over silicone control plates intraoperatively. The anterior tab of each implant was secured to the sclera using two 10–0 nylon sutures. The conjunctiva was reapproximated to the limbus using two 10–0 nylon wing sutures, after which proper implant and tube positioning in the anterior chamber were confirmed. Tobramycin and dexamethasone (MWI Animal Health) solution was applied to the eye at the conclusion of surgery, twice daily for 7 days and, once daily for 7 more days.

### 2.6 Bleb grading

The anterior segment (AS) and zone overlying the GDI were inspected twice daily for the first seven postoperative days, then daily until the end of each study. Then, 14‐ and 28‐days after surgery, IOP was measured prior to detailed examination under general anesthesia by a grader (IP or AB) masked to the type of implant used. Bleb morphology was graded during examination using the Indiana Bleb Appearance Grading Scale (IBAGS) which assigns numerical scores for height, extent, and vascularity based on standardized photos.^29^ Cross‐sectional AS optical coherence tomography (AS‐OCT) images of inflated blebs were obtained in some eyes using the Proveo 8 iOCT (Leica Microsystems Inc., Buffalo Grove, IL).

### 2.7 Bleb permeability measurement and fluorescein egress

Measurements were performed using constant‐flow perfusion of the GDI tube by anterior chamber cannulation, measuring the pressure within the device system at various flow rates. This differed from previously published method*s*,^16^, ^17^ in which constant pressure (12 mmHg) was maintained and the flow rate measured using devices with two tubes, one in the anterior chamber, and the other for measurement. Rabbits were placed under general anesthesia and given an intravenous infusion of 100 U/kg heparin (MWI Animal Health) prior to cannulation of the implant tube within the anterior chamber using a blunt‐tip 30 gauge needle. The needle was attached to a syringe micropump (Harvard Apparatus, Holliston, MA) and an inline pressure sensor (FISO Technologies, Quebec, Canada), allowing simultaneous infusion of a 0.1% sodium fluorescein solution (fluorescein sodium salt [Sigma, St. Louis, MO] in 0.9% sodium chloride) at physiologic flow rates (from 0.5 to 3 μl/min in 0.5 μl increments) and continuous pressure measurement (Figure 4a). Fluorescein solution was infused at increasing flow rates until bleb filling was visualized under an operating microscope and measured pressure reached a stable level, defined as pressure variation ≤±0.5 mmHg for 2 min. For all devices, pressure decay experiments were performed by stopping infusion, maintaining the seal between the cannula and the implant tube, and monitoring pressure decay over time. Decay was expressed as the ratio of pressure at each time point to the starting pressure at the cessation of flow (called the *p* ratio).

All eyes were observed throughout perfusion (typically 35–40 min) under cobalt blue light, and patterns of fluorescein movement into device tubes and reservoirs, beyond reservoir borders, and into surrounding tissues was described qualitatively. Of note, fluorescein perfusion was always performed immediately prior to sacrifice and enucleation, to avoid altering histopathology findings.

### 2.8 Histopathology

Rabbits were humanely sacrificed using a lethal injection of sodium pentobarbital 390 mg/ml + sodium phenytoin 50 mg/ml (MWI Animal Health); after enucleation, the superior and temporal aspect of each eye was labeled and all eyes were fixed in Bouin's solution (Sigma‐Aldrich, St. Louis, MO) for 2 days, then transferred to 10% neutral buffered formalin (Thermo Scientific, Kalamazoo, MI). Tubes were trimmed from the implants and tissue from the superotemporal quadrant (with devices in place) was embedded in paraffin for histopathological examination, sectioned laterally, and stained with hematoxylin/eosin or with Masson's trichrome. Sections were divided into quadrants (superior/inferior, conjunctival/scleral). Measurements of capsule thickness were made from the conjunctival side of the implant (Supplemental Figure S1). Slides were evaluated by a masked, trained pathologist (M. T.) and the zone of fibrous tissue between the implant and normal‐appearing conjunctiva was measured as the capsule. For capsular thickness measurements, at least 10 measurements were included per eye, representing a minimum of 5 different sections. In addition, the device area was qualitatively assessed for tissue inflammation adjacent to implants, cellular integration into device material, and undesirable folding of ePTFE devices, which are inherently more flexible than silicone.

### 2.9 Statistical analysis

All results are displayed as mean ± *SD*. For the IOP, bleb height, vascularity, and bleb extent analyses, two‐way analysis of variance (ANOVA) with Dunnett correction for multiple comparisons was used. Nested *t* tests were used for comparisons of capsular thickness of control and High implants. Two‐way ANOVA with Tukey correction for multiple comparisons was used for comparison of capsular thickness over High, Low, Filled, and Flat devices.

## 3 RESULTS

### 3.1 Bleb morphology

**FIGURE 2:**
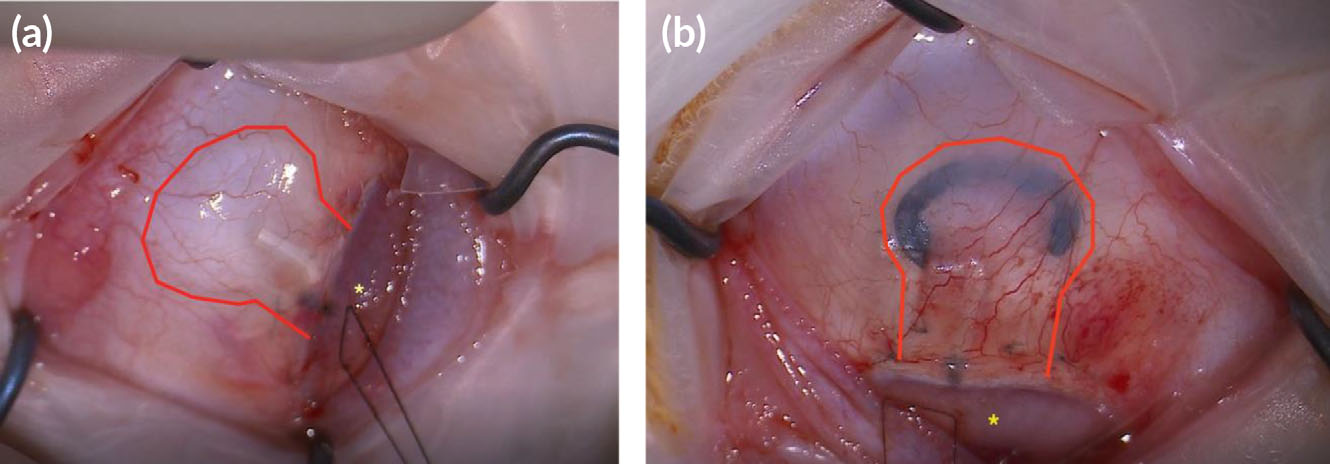
Pictures of implants after placement. Pictures of (a) control and (b) expanded polytetrafluoroethylene (ePTFE) implants after placement in rabbit eyes (outlined in red; yellow * denotes cornea; *High* implant edge inked for visualization)

**FIGURE 3:**
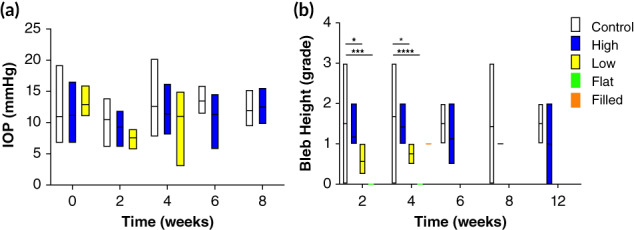
Clinical characterization of implant healing (a) intraocular pressure (IOP) and (b) Indiana Bleb Appearance Grading Scale (IBAGS) bleb height grading of implants through 8 weeks after placement. Only control and *High* implants were followed past 4 weeks after placement. *p < .05, **p < .01, ****p < .0001

Implant blebs, shown in Figure 2a,b, were graded for height, extent, and vascularity using the IBAGS tool for all devices at 2 and 4 weeks after placement, and at 6, 8, and 12 weeks for control and High devices.^29^ Mean grades for bleb extent and vascularity did not differ significantly among devices, but there were significant differences in bleb height between control devices and some ePTFE devices (Figure 3b). At both 2 and 4 weeks after implantation, control device blebs (IBAGS score 1.50 ± 1.05 and 1.68 ± 0.77, respectively) were significantly higher than Low (0.56 ± 0.38, *p* = .05 at 2 weeks and 0.75 ± 0.29, *p* = .04 at 4 weeks) and Flat (0 ± 0 at 2 and 4 weeks, *p* = .0005) blebs. There was no significant difference between control and High device bleb heights at 2 and 4 weeks (*p* = .65 and *p* = .69, respectively) or between control and Filled device bleb heights at 2 and 4 weeks (*p* = .51 and *p* = .19, respectively). High blebs were significantly higher at 2 and 4 weeks than Low (*p* = .05 and *p* = .03, respectively) and Flat (*p* = .0005 and *p* < .0001, respectively). At 6 and 12 weeks, control and High devices were not significantly different in height (Figure 3b).

### 3.2 Intraocular pressure

IOPs were measured for all implant models for 1 month after implantation and for 12 weeks for devices with tubes open to the anterior chamber (Figure 3a). IOPs did not decrease significantly from baseline in any group and did not differ significantly among implant models.

### 3.3 Tissue integration and capsular thickness

**FIGURE 4:**
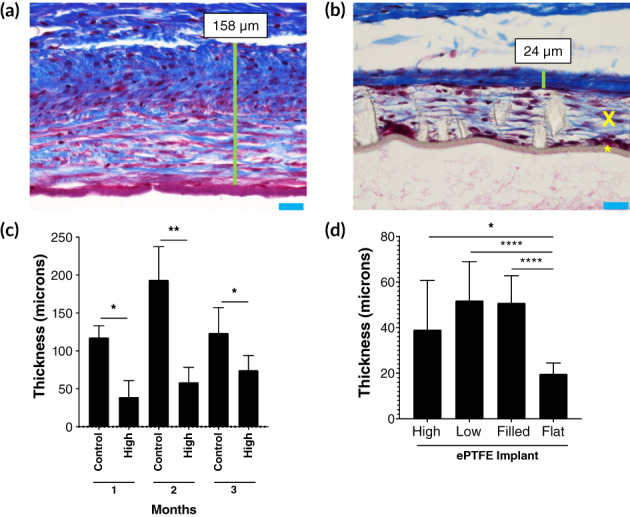
Capsule histopathology and thickness. Masson trichrome stained cross‐sections of control (a) and *High* (b) plates show capsule thickness (green line) and cellular integration into expanded polytetrafluoroethylene (ePTFE) implants with black asterisk showing the impermeable ePTFE layer and the black “X” showing the layer of cellular integration (blue scale bar = 25 μm; yellow arrowhead = macrophage, asterisk = giant cell, arrows = fibroblasts). (c) Capsular thickness of blebs around *High* and control implants 1, 2, and 3 months after implantation (*n =* 4 per group). (d) Capsular thickness of blebs around each ePTFE implant type at 1 month (*n* = 4 per group). *p < .05, **p < .01, ****p < .0001

Histopathological examination of implants demonstrated cellular integration into ePTFE material and reduced capsular thickness around the ePTFE versus the control devices. Cellular integration into the outer layer, but not into or through the inner layer, of the ePTFE devices was apparent microscopically, as was the formation of a fibrous capsule outside the integrated cellular layer (Figure 4a,b). Control plates did not show integration and the blebs surrounding the silicone plates exhibited dense fibrous capsules that were significantly thicker than those around the High implants at 1 month (117.5 ± 48 μm for control vs. 40.2 ± 32 μm for High, *p* = .048), 2 months (193.6 ± 95 μm for control vs. 61.4 ± 53 μm for High, *p* = .0086), and 3 months (129.7 ± 56 μm for control vs. 74.3 ± 31 μm for High) after implantation (Figure 4c; Supplemental Table S1). While there was a trend toward increasing High capsular thickness between 1 and 3 months, this change was not significant (*p* = .18). AS‐OCT images of control and High implants qualitatively suggest greater capsule density over control devices (Supplemental Figure S2).

To assess the effect of bleb height on capsular thickness in ePTFE devices, we compared ePTFE devices with identical footprints but differing heights when inflated: High, Low, and Flat (Figure 4d). One month after placement, capsules were significantly thicker over both the High (40.2 ± 32.3 μm, *p* = .01) and the Low (50.7 ± 21 μm, *p* < .0001) implants than the Flat implant (19.5 ± 8.4 μm) that was not connected to the anterior chamber (Supplemental Table S1). There was no significant difference in capsular thickness between High and Low implants (*p* = .18), both of which were able to inflate with AH exposure, though to different degrees.

Finally, we examined the effect of AH flow on capsular thickness by comparing capsular thicknesses around the High and Low implants to those around ePTFE implants designed to have an inflated contour in the absence of AH or any fluid flow (Filled). There were no significant differences between capsular thicknesses around the High (*p* = .25) or the Low (*p* = .99) implants compared to the Filled implant (50.9 ± 29 μm). Notably, the Filled capsule was significantly thicker than Flat, despite a lack of AH flow through both implants (*p* < .0001) (Figure 4e).

### 3.4 Permeability assessment under constant‐flow perfusion

**FIGURE 5:**
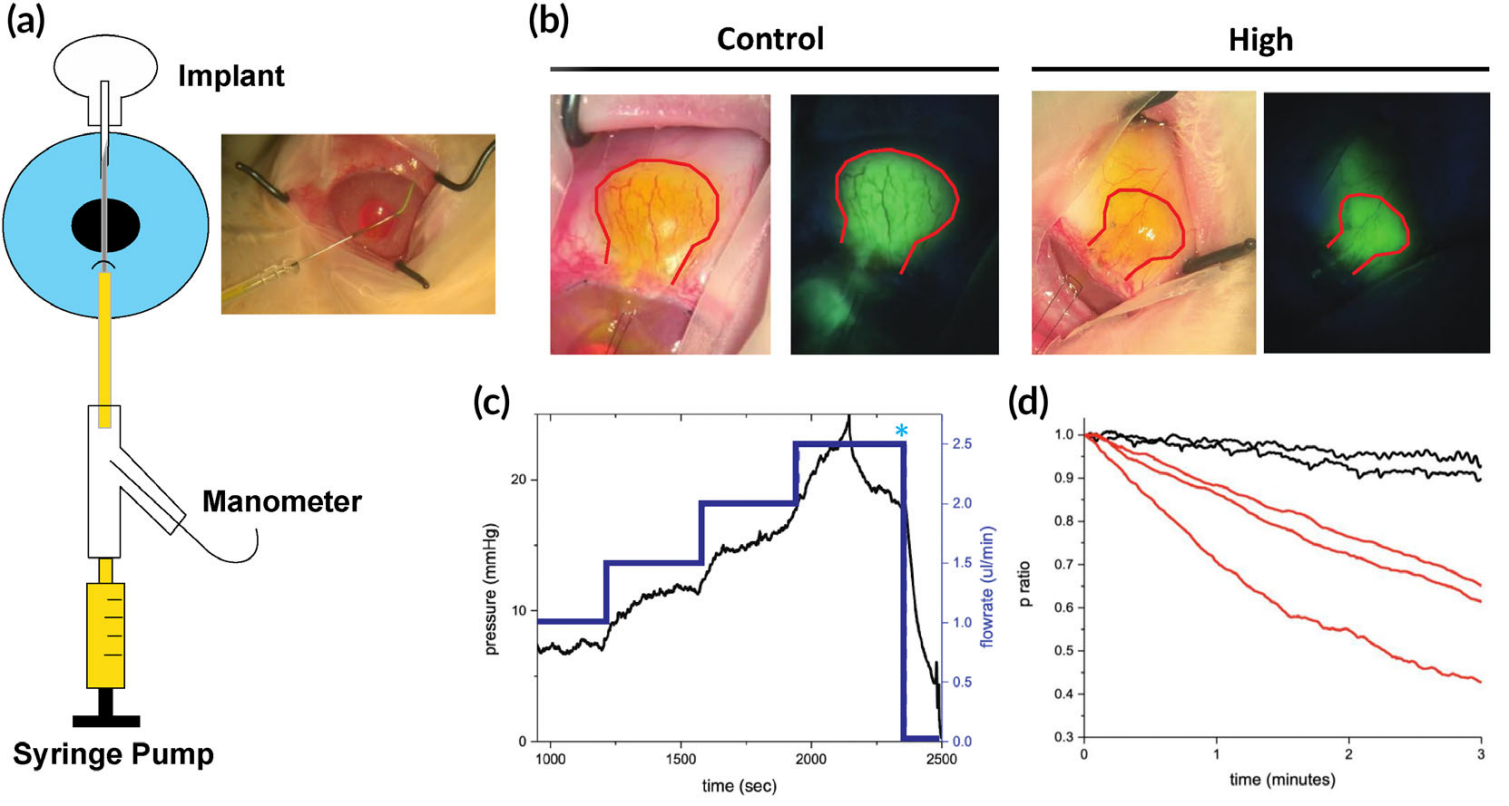
Bleb permeability studies. (a) Schematic of apparatus for studying bleb permeability (left) and picture of anterior chamber cannulation of tube (right). (b) Images of control and *High* plates after fluorescein infusion (29–30 μl infused over 36–40 min) (plate border highlighted in red) show extension of fluorescein flow beyond the plate. (c) Permeability studies of control (left) and *High* (right) implants demonstrate absence of steady‐state flow and pressure decay in control implant. (d) Decay ratio (*p* ratio) of control (black) and *High* implants (red), measured after stopping fluorescein irrigation

The reduced capsular thickness of blebs associated with ePTFE implants versus controls suggested that ePTFE‐associated blebs could be more permeable to AH. To test this hypothesis, we performed fluorescein studies to visualize and measure flow through the device zone over ePTFE and control implants following implantation. The tube of each implant was directly cannulated in the anterior chamber and fluorescein was infused at physiologic rates in steps from 0.5 to 3 μl/min using a syringe pump connected to a manometer (Figure 5a).^17^

Patterns of bleb fluorescence differed significantly between ePTFE and control implants (Figure 5b). The region of visible fluorescein around the plate portion of control devices was sharply demarcated and did not extend beyond the implant's footprint (*n* = 5). In contrast, fluorescence around ePTFE implants open to AH flow (High and Low) extended beyond the implants' margins toward the fornix in the subconjunctival space (*n* = 5). This suggested that blebs produced around control devices had limited permeability compared to blebs around ePTFE implants. To estimate bleb permeability quantitatively, High and control implants were tested 1 month after implantation using constant‐flow perfusion. In High blebs, steady‐state pressures were obtained at each flow rate from 1.0 to 2.5 μl/min (Figure 5c). By contrast, control blebs filled to a maximum volume, after which additional perfusion produced continuous rapid pressure increases that did not reach a steady state, even at the lowest flow rate (0.5 μl/min). Ultimately, in control blebs, rising pressure caused fluorescein back‐flow into the anterior chamber, presumed to be along the outside of the tube channel (Figure 5b).

Steady‐state pressures were obtained on perfusion of High (Figure 5c) but not control devices. It was additionally observed that when perfusion was stopped (Figure 5c, blue asterisk) there was a rapid pressure decay in High implants despite continued watertight cannulation, which was not seen in control implants. We therefore chose to further characterize this pressure decay in High and control implants. For both control and High devices, after the highest perfusion rate was reached, fluorescein infusion was stopped and pressure was monitored continuously while the seal between the irrigation cannula and the tube was maintained. Pressure remained high in blebs overlying control devices, with minimal detectable decay, or decrease in “*p* ratio” (Figure 5d). In ePTFE‐associated blebs, a rapid pressure decay (*p* ratio decrease) was observed over the course of several minutes (Figure 5d). The rate of decay shown in Figure 5d was notably higher in ePTFE‐associated blebs (red lines) compared to control blebs (black lines). Due to challenges associated with prolonged perfusion (involuntary animal movement, cannula dislocation, fluorescein back‐flow from control blebs) complete *p* ratio curves obtained were too few for statistical analysis (five total; two control, three ePTFE), but observed decay behavior was consistent with that described for all devices.

### 3.5 Complications

Three implants, all control type, were excluded from the study data due to complications. One had an area of conjunctival erosion noted on postoperative Day 40 and it was explanted. A second control was explanted due to exposed implant on Day 41. The third implant had separation of tube and plate noted immediately prior to sacrifice.

Four minor clinical complications occurred, one control and three ePTFE, but their data could be included. One control implant had a swollen painful eyelid of unknown etiology on Day 3. The pain was addressed with a single dose of systemic buprenorphine and swelling and discomfort rapidly resolved. A High implant eye was treated for anterior chamber inflammation (fibrin) of unknown etiology on Days 38–40 with tobramycin/dexamethasone eye drops. The inflammation was not associated with implant exposure or infection and resolved with treatment, so the rabbit was continued in the study. Two implants, one High, and one Low had exposed anterior tabs at Day 14. As there was no AH leakage of AH, the tabs were trimmed flush.

## 4 DISCUSSION

Studies described here reveal four main findings: (a) the bleb capsule associated with ePTFE implants was significantly thinner compared to silicone control at 1, 2, and 3 months after implantation; (b) increased bleb height among ePTFE implants led to increased capsular thickness and (c) this effect was independent of AH exposure; and (d) finally, blebs associated with ePTFE implants showed greater fluorescein permeability than silicone controls.

**FIGURE 6:**
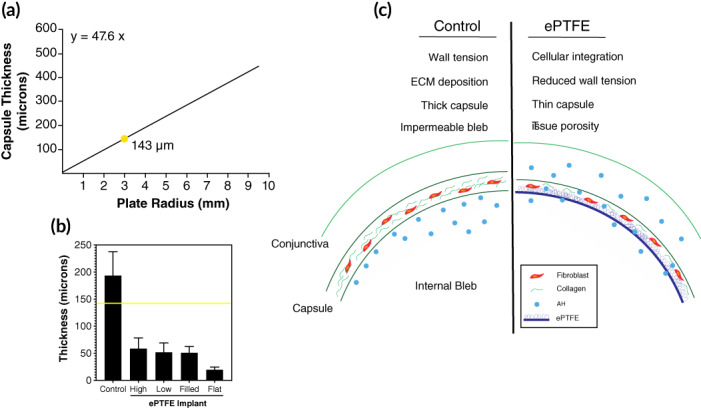
Expanded polytetrafluoroethylene (ePTFE) decouples plate radius and capsule thickness. (a) The predicted capsule thickness for the experimental plates (3 mm radius) is 143 μm.^15^ (b) Capsule thickness of control and ePTFE implants in relation to the predicted value (yellow line). (c) Conceptual model of reduced capsular thickness and increased permeability through tissue integration into a porous ePTFE layer

The fibrous capsule over ePTFE implants was significantly thinner than that over the control device. Wilcox et al investigated the relationship between bleb capsular thickness and implant device radius in rabbits for several GDIs, concluding that device radius related to capsule thickness in a linear fashion (Figure 6a).^14^, ^15^ These findings supported the hypothesis that, as increasing device size leads to formation of larger, higher blebs, wall tension increases and the capsule thickens. They further hypothesized that increased capsular thickness was due to fibroblast exposure to greater mechanical stresses within a bleb in which tension was determined both by geometry and IOP.^30^ This hypothesis is supported by the current understanding in mechanobiology that fibroblasts respond to tensile, compressive, and shear stresses and strains with a fibrotic response.^31^ In our studies, blebs over control devices had mean thickness (118–193 μm) consistent with predictions from Wilcox et al. for plates with major axis of 3 mm (143 μm) (Figure 6a). In contrast, ePTFE implants with the same dimension had capsules that were significantly thinner than controls (Figure 6b).

Why was bleb thickness uncoupled from implant geometry by ePTFE? One possible explanation could be the unique biointerface created by tissue integration into the outer layer of our ePTFE device (Figure 6c). Integration into ePTFE could decrease capsule tension experienced by cells in the bleb through load sharing with the implant itself. Furthermore, the ePTFE could provide topographic cues that alter orientation of collagen deposition and capsular thickness.^32^ If such decreases in capsular thickness are associated with increased bleb permeability in further clinical experiments, ePTFE implants may represent a means to improve human glaucoma surgery.

Our studies showed no difference in bleb capsule thickness whether devices of the same geometry were exposed or not exposed to AH. This result was surprising, as multiple profibrotic factors have been identified within AH of glaucomatous eyes, particularly in the early postoperative period. The presence of some of these factors has been associated with surgical failure.^11^, ^12^, ^13^, ^14^, ^15^ Several hypotheses could contribute to our failure to detect a role for exposure to AH. First, the interaction of ePTFE with cells during the healing process could mitigate the effect of cytokines on development of the fibrous capsule. Second, profibrotic factors in AH of non‐glaucomatous rabbits could be below threshold levels that influence fibrosis; however, rabbit eyes are notorious for a vigorous healing response. Third, Wilcox et al showed prolonged aqueous flow was associated with increased bleb permeability in monkey eyes.^30^ Therefore, there are potentially conditions under which bleb perfusion with AH enhances rather than inhibits bleb permeability. Finally, the putative AH effect could be primarily mediated by the height and tension of the capsule. These results highlight the importance of bleb geometry as a modifiable factor that can determine capsule thickness and, potentially, permeability to AH outflow in glaucoma surgery.

A major determinant of safe, successful IOP reduction in glaucoma surgery is a durable, sufficiently permeable bleb. Several mechanisms of bleb permeability have been proposed, including absorption into conjunctival vasculature, clearance via conjunctival lymphatics, and diffusion through tissue interstitium.^6^ While we did occasionally observe fluorescein drainage in a pattern consistent with lymphatic clearance (Supplemental Figure S3), the main pattern of clearance observed was diffuse, within the conjunctival tissue, and consistent with interstitial flow toward the fornix. The gold standard for determining bleb permeability should be direct measurement of fluid flow. We noted poor flow in control blebs as demonstrated by (a) an inability to achieve steady‐state flow on fluid challenge, (b) absence of pressure decay after stopping fluid flow, and (c) confinement of fluorescein to the bleb overlying the implant plate. In contrast, ePTFE‐associated blebs (a) reached a steady‐state outflow at physiologic flow rates, (b) underwent rapid pressure decay on stopping fluid flow, and (c) allowed extension of fluorescein beyond the implant border.

Several bleb features may contribute to permeability, including capsular thickness and surface area. Prior permeability studies using a different method from ours to examine non‐ePTFE implants in rabbits found very low permeability in healed implant blebs that decreased both with increased thickness and with fluid challenge; this relationship is not fully understood.^16^, ^17^, ^28^ In addition to capsule thickness, bleb surface area also determines permeability.^13^ In our studies, all implants had identical footprints and their overlying blebs were generally restricted to their footprint. Since bleb heights were the same in ePTFE (inflatable) devices and controls, the internal surface area for AH diffusion was similar. Yet, ePTFE devices had better permeability, indicating that their thinner capsules were more permeable either due to thinness alone, or due to favorable characteristics of the interaction of ePTFE membranes with the capsule wall.

ePTFE has been evaluated previously in glaucoma surgery.^33^, ^34^, ^35^, ^36^, ^37^, ^38^, ^39^ The Ahmed implant was modified with an outer ePTFE membrane to create the PRIME‐Ahmed, which showed increased resistance to fluid outflow in vitro, but created a thinner capsule in rabbit eyes when compared to unmodified Ahmed implants.^34^, ^36^ Of note, blebs associated with the PRIME‐Ahmed showed a greater degree of vascularization, collagen disorganization, and chronic inflammation compare to control implants; these features were not noted in our histopathological exams. More recently, an ePTFE‐based membrane‐tube‐type glaucoma shunt device was described as having good biocompatibility and IOP reduction in rabbit eyes, as well as IOP reduction in patients with refractory glaucoma.^37^, ^40^, ^41^ Our implants differed from both previous devices in that the microstructure of the unique, bilayered ePTFE used here was chosen and manufactured specifically for this application. We further add to previous studies by (a) including silicone controls with matching footprints to ePTFE devices, (b) varying device height to clarify the effect of device geometry on capsule thickness, (c) evaluating the independent contribution of AH flow to capsule thickness, and (d) evaluating bleb permeability.

These studies have several limitations. First, while NZW rabbits are an established model in glaucoma surgery, there are aspects of their anatomy and fibrotic response that do not mirror human eyes. In NZW rabbits, implants needed to be placed significantly closer to the limbus than would be traditionally done in humans to avoid venous sinuses within the orbit. Additionally, rabbit filtering surgeries typically fail within 4 weeks,^42^ which is significantly more rapid than in the average human eye. Our surgeries allowed for immediate, unrestricted flow from the anterior chamber into the subconjunctival space; while this approach is tolerated in rabbit eyes,^43^ it does not recapitulate surgery in humans, in which early postoperative flow restriction is common with some implants. Our approach, however, could potentially expose the subconjunctival space to more inflammatory cytokines in the AH in the immediate postoperative period. Our sample size was not powered to detect a change in IOP in response to surgery. The exclusion of IOP as a primary outcome was based on previous studies that did not observe IOP reduction after GDI placement in normotensive eyes;^30^ to detect IOP change would have required not only a larger sample but also a protocol to induce ocular hypertension in rabbit eyes. Finally, we followed implants through 3 months after placement. While this time period was described previously as sufficient for bleb stabilization,^30^, ^44^ future studies should follow implants for longer durations.

## 5 CONCLUSIONS

These studies demonstrated thinner capsule formation and increased permeability of ePTFE implants compared to silicone controls in rabbit eyes, which was sustained at 3 months. Capsule geometry—and not AH flow—determined capsule thickness in this model as well. These findings could aid in the future design of implants for glaucoma surgery.

## 6 CONFLICT OF INTEREST

This work was sponsored by a grant from W.L. Gore & Associates, Inc., Newark, Delaware. P. R., J. T., and M. T. are employees of the research sponsor, W.L. Gore & Associates, Inc. and received no additional compensation, funding, or support in connection with the study. All other authors are paid consultants to W.L. Gore & Associates, Inc.

## Supporting information


**Table S1**. Supporting Information.

**Supplemental Figure S1 Capsule thickness measurement**. TRM staining of *High* implant (blue scale bar = 1,000 μm). Each section was divided into quadrants (Q1‐Q4). Statistical analyses were performed for capsular thickness measurements taken from conjunctival quadrants (Q1 and Q4).

**Supplemental Figure S2 Capsular OCT images**. Cross‐sectional OCT images taken 2 months after implantation through the widest portion of control (A) and *High* (B) implants parallel to the limbus (scale bar = 1 mm). Conjunctival side is at the top of the slide and scleral side at the bottom. Dimensions of control plate and *High* are highlighted with dashed line.

**Supplemental Figure S3 Presumed lymphatic bleb outflow**. Intraconjunctival fluorescein pattern consistent with lymphatic outflow (arrows) from implant bleb (asterisk).
