# Supplementary material for: A novel bilayered expanded polytetrafluoroethylene glaucoma implant creates a permeable thin capsule independent of aqueous humor exposure
Source: Bioeng Transl Med. 2020 Aug 22;6(1):e10179. doi: 10.1002/btm2.10179 (PMC7823119; doi:10.1002/btm2.10179)
Supplement: Supplementary file 1 — Table S1. Supporting Information. [file BTM2-6-e10179-s001.docx]

**Supplemental Table 1.**

|  | **Device Name** | **Timepoint (months)** | **N** | **Capsule Thickness (SD)**  **(µm)** | ***p*-value** |
| --- | --- | --- | --- | --- | --- |
| a | Control | 1 | 4 | 117.5 (48) | a-b (0.048) |
| b | High | 1 | 3 | 40.2 (32) | c-d (0.0086) |
| c | Control | 2 | 4 | 193.6 (95) | e-f (0.02) |
| d | High | 2 | 4 | 61.4 (53) | b-g (0.18) |
| e | Control | 3 | 4 | 129.7 (56) | b-h (0.01) |
| f | High | 3 | 4 | 74.3 (31) | b-i (0.25) |
| g | Low | 1 | 4 | 50.7 (21) | g-h (<0.0001) |
| h | Flat | 1 | 3 | 19.5 (8.4) | g-i (0.99) |
| i | Filled | 1 | 4 | 50.9 (29) | h-i (<0.0001) |
