# Supplementary figures and images for: A novel bilayered expanded polytetrafluoroethylene glaucoma implant creates a permeable thin capsule independent of aqueous humor exposure
Source: Bioeng Transl Med. 2020 Aug 22;6(1):e10179. doi: 10.1002/btm2.10179 (PMC7823119; doi:10.1002/btm2.10179)

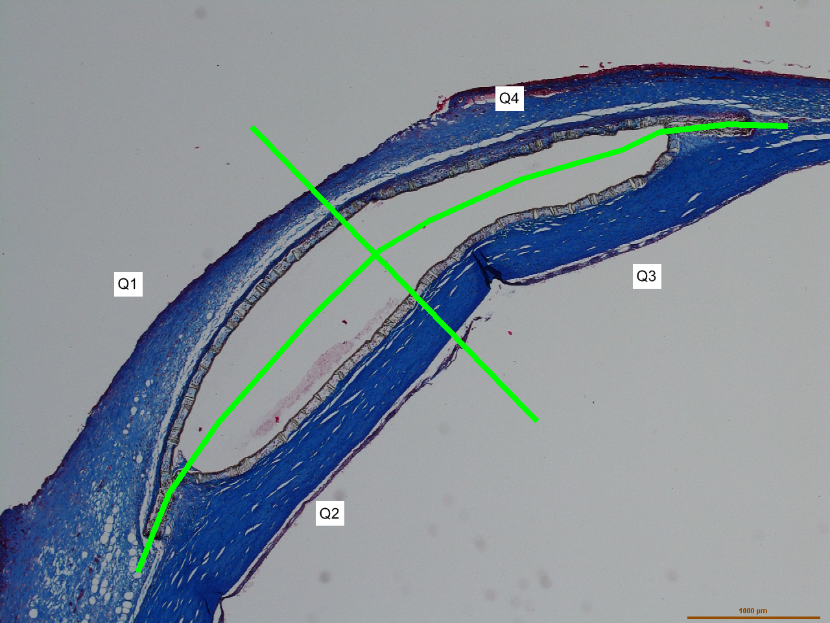

Supplement: Supplementary file 2 — Supplemental Figure S1 Capsule thickness measurement. TRM staining of High implant (blue scale bar = 1,000 μm). Each section was divided into quadrants (Q1‐Q4). Statistical analyses were performed for capsular thickness measurements taken from conjunctival quadrants (Q1 and Q4). [file BTM2-6-e10179-s002.docx]

**A**


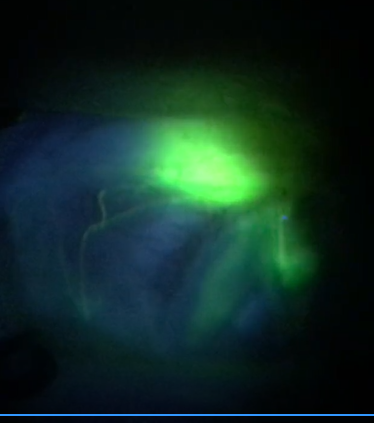


*

Supplement: Supplementary file 4 — Supplemental Figure S3 Presumed lymphatic bleb outflow. Intraconjunctival fluorescein pattern consistent with lymphatic outflow (arrows) from implant bleb (asterisk). [file BTM2-6-e10179-s004.docx]
